# The road to refractory graft-versus-host disease is paved with good intentions

**DOI:** 10.1172/JCI177728

**Published:** 2024-04-01

**Authors:** Daniel North, Ronjon Chakraverty

**Affiliations:** MRC Weatherall Institute of Molecular Medicine, University of Oxford, Oxford, United Kingdom

## Abstract

Refractory acute graft-versus-host disease (GVHD) occurs when the immune injury exceeds the capacity of injured tissues to regenerate and repair. While glucocorticoids have been used for decades to treat GVHD, Arnhold, Chang, and colleagues in this issue of the *JCI* question whether this approach can in fact be counterproductive. Using in vivo experimental models of GVHD and in vitro intestinal organoids, the study authors show that glucocorticoid exposure directly impeded small intestinal epithelial proliferation and survival, thus preventing the resolution of injury. These findings suggest that future treatment approaches for acute GVHD should include measures to reduce immune reactivity as well as interventions to actively promote tissue resilience.

## Glucocorticoids in GVHD

Acute graft-versus-host disease (GVHD) remains one of the major barriers to successful allogeneic hematopoietic stem cell transplantation (1). While glucocorticoids have been the standard first-line therapy for over 40 years (2), early treatment resistance is observed in approximately 50% of patients and is associated with poor clinical outcomes, especially in patients with intestinal GVHD (1). To date, clinical trials testing the use of additional small molecules or biologicals given in combination with glucocorticoids at the onset of acute GVHD have consistently failed to improve survival (3–5). Although the use of ruxolitinib as salvage for steroid-refractory acute GVHD is an important advance (6), the failure rate remains substantial, and patients with lower intestinal GVHD in particular fare poorly (7). Ever-increasing immune suppression has been trialed and does not work (8). So why are we still failing to make progress? Elegant research from the laboratory of Alan Hanash, reported in this issue of the *JCI* (9), now provides a potential answer and makes uncomfortable reading for clinicians treating GVHD. Are glucocorticoids actually driving refractory intestinal GVHD?

Arnhold et al. focused on the effect of glucocorticoids on the small intestinal epithelia in steady state and following genotoxic or immune injury (9). High rates of cell turnover in the steady state are required to maintain intestinal epithelial homeostasis (10), a demand met by the immense regenerative capacity of adult intestinal stem cell (ISC) populations (11). Following irradiation-induced injury, both surviving stem cells and more committed transit-amplifying cells within crypt-proximal regions zone of the villus undergo a proliferative response to ensure rapid reepithelialization (12). In GVHD, sensitivity of leucine-rich repeat–containing GPCR 5 (Lgr5)^+^ ISCs to IFN-γ–mediated apoptosis (13), in combination with derangement of stem cell–supportive niches (14), drastically reduces the capacity for regeneration. Glucocorticoids have generally been regarded as a means of curtailing this immune response, but the broader effects of the drug on the tissues themselves have been underinvestigated.

After first demonstrating glucocorticoid receptor (GR) expression in mouse and human ISCs and enterocyte progenitors, the Hanash group used a combination of in vivo and intestinal organoid modeling to test how these cell populations were affected by glucocorticoids (9). While short-term exposure of mice to glucocorticoids in steady state did not cause any observable clinical effect, the authors noted changes to ileal crypt and villous architecture consistent with reductions in epithelial cell proliferation, a finding supported by reduced Ki67 protein expression. Intestinal organoids derived from mice exposed to glucocorticoids were smaller than those in control mice. In vitro exposure to glucocorticoids resulted in GR-dependent inhibition of Lgr5^+^ ISC proliferation. Genes encoding cell-cycle–promoting cyclins were also reduced, whereas expression of the *Cdnka* gene, which encodes the cell-cycle inhibitor p21, was increased. The authors then examined the effect of glucocorticoids following irradiation, and here the effects were more nuanced. When administered early (from 24 hours) following irradiation, glucocorticoids were protective in preserving ileal crypts in vivo and improved organoid viability in vitro. Although not tested in Arnhold et al., it is conceivable that early corticosteroid repression of proliferation following irradiation protected ISCs by preventing checkpoint adaptation, the process of precocious cell-cycle progression with incomplete repair of DNA damage, previously shown to mediate ISC loss following radiation injury (15). In contrast, when administered later following irradiation (from 72 hours), this protective effect was lost, and glucocorticoids induced an epithelial proliferative defect and loss of crypts as observed in steady state. In the context of GVHD, in which recovery requires epithelial regeneration in response to the composite of genotoxic and immune injury, exogenous glucocorticoids countered the proliferative response (9). Using an organoid system to model the IFN-γ–mediated injury that is stereotypic of GVHD (13), glucocorticoids induced the expression of proapoptotic genes and reduced cell survival (9).

## The effects of IL-22

Arnhold, Chang, and authors reasoned that the combination of glucocorticoids with approaches that directly prevent ISC apoptosis would improve intestinal epithelial recovery in gut GVHD (9). Following irradiation, myeloid cells recruited to the intestine secrete IL-23, a cytokine that triggers local type 3 innate lymphoid cell (ILC3) to generate local IL-22; this latter cytokine then acts directly on ISCs and transits amplifying cells to promote survival via a STAT3-dependent mechanism (16). This recovery mechanism is deficient in GVHD, partly through the loss of ILC3 cells and a consequent loss of IL-22 (16). To test whether IL-22 would promote epithelial regeneration, mice with GVHD were treated with glucocorticoids alone or in combination with F-652, a recombinant human IL-22 dimer and Fc fusion protein. Combined treatment better preserved proliferative regeneration, epithelial integrity, and ISC numbers when compared with glucocorticoids given alone. Using the organoid system, the authors were able to show that IL-22 was capable of inducing STAT3 activation, even in the presence of glucocorticoids. From a translational perspective, the phase II trial of F-652 in combination treatment with glucocorticoids for acute intestinal GVHD has recently demonstrated the safety of this approach (17), assuaging concerns that IL-22 might amplify some components of immune injury. There is need now for randomized, controlled trials of F-652 to test efficacy.

## Effect of glucocorticoids on tissue tolerance

**Figure 1:**
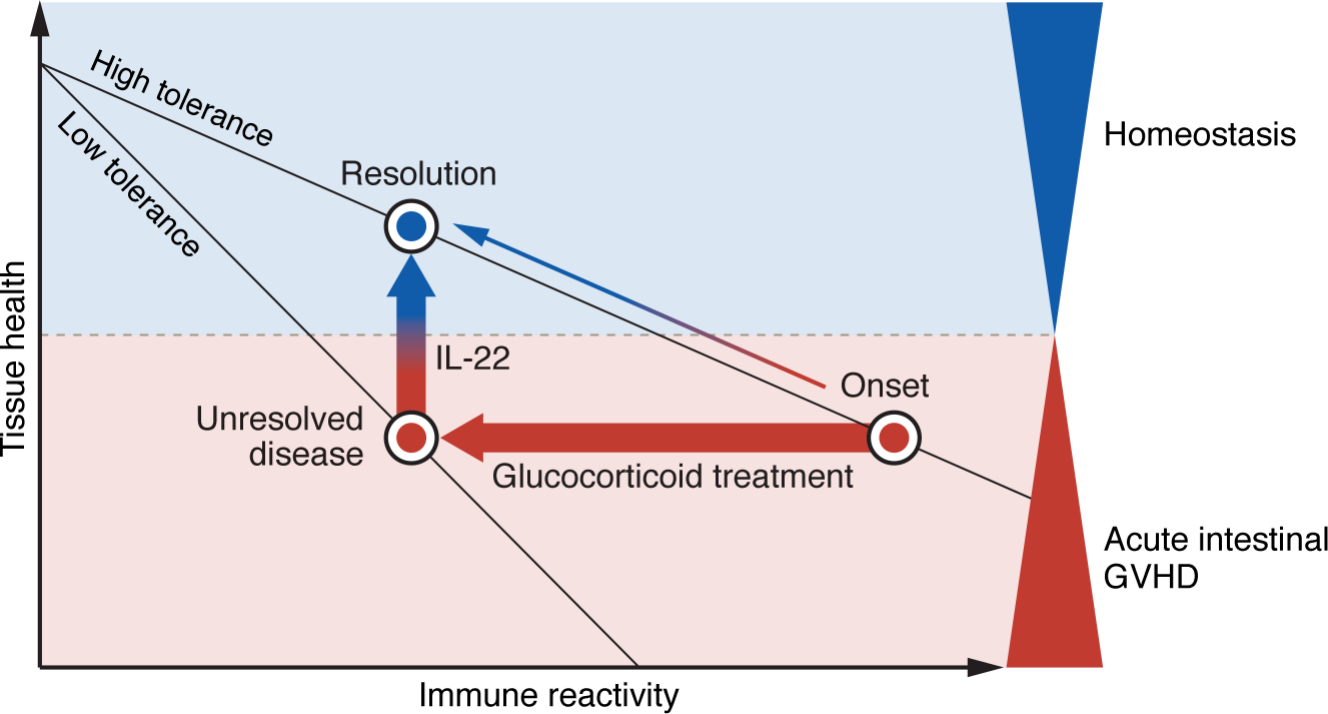
Glucocorticoids impair tissue tolerance in intestinal GVHD. The relationship between tissue homeostasis and acute intestinal GVHD can be modeled in a linear graph as tissue health versus immune reactivity, in which the degree of tolerance is reflected in the steepness of the slope. In the absence of immune injury, tissue health corresponds with homeostasis. Tissues with high tolerance display a shallow slope and are more likely to reach resolution with treatment. Conversely, tissues with low tolerance show a steep slope and are more likely to remain in an unresolved disease state despite treatment. At the onset of acute intestinal GVHD, inhibition of immune reactivity in tissues with high tolerance may permit the resolution of disease, provided baseline tissue tolerance remains intact. However, if glucocorticoid treatment not only reduces immune reactivity but also reduces tissue tolerance, GVHD will remain unresolved. Notably, the promotion of epithelial regeneration by IL-22 can offset glucocorticoid mediation reductions in tissue tolerance, thus facilitating a return to homeostasis and health. Diagram adapted from Wu and Reddy (25).

In seeking to interpret the Arnhold et al. findings, we find it useful to apply the conceptual framework of tissue tolerance introduced by Reddy and colleagues (18), who repurposed ideas derived from models of disease tolerance to pathogens (19) (Figure 1). According to this concept, important components of tissue tolerance include cellular resistance to apoptosis in target organs and the regenerative or repair capacity to restore tissue homeostasis after immune injury. It is thus possible to conceive of differing levels of tissue tolerance, which define different tissue responses to injury. In the setting of a genotoxic insult alone, such as irradiation or cytoreductive chemotherapy, intestinal injury is tolerated because of the huge regenerative capacity of surviving ISCs and the plasticity of enterocyte precursors, which regain stem cell–like properties in response to local WNT signaling (12). In the context of immune injury in GVHD, combined genotoxic and immune injury reduces the regenerative capacity by targeting ISCs and disrupting critical stromal-epithelial-immune interactions that promote epithelial integrity (reviewed in ref. 20). The aim of immune-suppressive therapy is to block alloreactivity and promote counterregulatory immune responses within tissues to a level that preserves sufficient regenerative capacity and recovery toward health. However, although glucocorticoids promote immune tolerance, they also directly reduce tissue tolerance by further impeding the capacity for epithelial regeneration. Although IL-22 has little effect on reducing immune reactivity (21), it can improve tissue tolerance. Thus, combined treatment with IL-22 and glucocorticoids has the potential to reduce immune reactivity and increase tissue tolerance simultaneously (Figure 1), permitting disease resolution.

## Clinical implications

A key issue for future studies is to identify the molecular mechanisms whereby glucocorticoids impair intestinal epithelial cell proliferation and the extent to which these intersect with pathways regulated by IL-22. While glucocorticoids can antagonize STAT3 (22), this interaction is complex, and it remains unclear whether this pathway is critical to glucocorticoid inhibition of epithelial regeneration. An important therapeutic concern is whether ruxolitinib-mediated antagonism of the STAT3 pathway (23) may also negatively affect epithelial regeneration, a finding suggested by the organoid studies in Arnhold et al. (9), even though the same drug can prevent ISC apoptosis in GVHD by inhibiting JAK1/STAT1 signaling (13). These data highlight the need for investigators to carefully consider how individual agents currently in use or being trialed for the treatment of GVHD affect not only immune tolerance but also the capacity of tissues to regenerate and repair. Rational use of combination therapies directed at reducing immune injury while promoting increased tissue tolerance may ultimately be the answer, as suggested in the study by Arnhold et al.

The elephant in the room, of course, is: What should clinicians do when they are facing a patient with steroid-refractory intestinal GVHD? After all, gut biopsies from such patients are reported to show features consistent with regenerative failure (24). Therefore, should we stop the steroids? We do not yet have an evidenced-based answer to this question, but there is an important ongoing need for deep experimental interrogation of mechanisms underpinning the failure of steroids and other drugs in patients with GVHD. This research will require intensive effort within trials or observational studies that sample affected tissues, with the goal to define and validate measures of regenerative capacity and repair, identify cellular and molecular mechanisms underpinning a lack of response, and test approaches that actively promote tissue tolerance.

## Version 1 04/01/2024

Electronic publication
